# NMR-based metabolomic profile of hypercholesterolemic human sera: Relationship with *in vitro* gene expression?

**DOI:** 10.1371/journal.pone.0231506

**Published:** 2020-04-16

**Authors:** Manuela Grimaldi, Angelica Palisi, Carmen Marino, Paola Montoro, Anna Capasso, Sara Novi, Mario Felice Tecce, Anna Maria D’Ursi

**Affiliations:** Department of Pharmacy, University of Salerno, Fisciano, Italy; National Institute for Medical Research, Medical Research Council, London, UNITED KINGDOM

## Abstract

Hypercholesterolaemia is considered an important cause of atherosclerotic cardiovascular disease. In a previous investigation, we demonstrated that cultured hepatoma cells treated with hypercholesterolaemic sera compared with cells treated with normocholesterolaemic sera show overexpression of mRNAs related to mitochondrial 3-hydroxy-3-methylglutaryl-coenzyme A synthase (HMGCS2). In the present work, using an NMR metabolomic analysis, we demonstrate that the hypercholesterolaemic blood sera previously used to treat cultured hepatoma cells are characterized by a metabolomic profile that is significantly different from the normocholesterolaemic sera. Acetate, acetone, 2-hydroxybutyrate, cysteine, valine, and glutamine are the metabolites distinguishing the two groups. Abnormalities in the concentrations of these metabolites reflect alterations in energy-related pathways, such as pantothenate and CoA biosynthesis, pyruvate, glycolysis/gluconeogenesis, the citrate cycle, and ketone bodies. Regarding ketone bodies, the pathway is regulated by HMGCS2; therefore, serum samples previously found to be able to increase HMGCS2 mRNA levels in cultured cells also contain higher amounts of the metabolites of its encoded enzyme protein product.

## Introduction

Excessive serum cholesterol concentration is considered an important cause of atherosclerotic cardiovascular disease [1]. Cholesterol is deeply involved in numerous molecular mechanisms leading to vascular damage; therefore, its concentration is widely used as a diagnostic clinical parameter to assess atherosclerotic disease and risk [2]. Most importantly, backgrounds of hypercholesterolemia can be mainly separated between those deriving from lifestyle, with a significant role of the quality and quantity of nutritional intake, and, much less frequently, those deriving from genetic factors [3].

Nevertheless, cholesterol concentration is only one element of a general metabolic condition that includes, but is not limited to, lipidic components. This metabolic change is generally known to be the causal pathogenic mechanism resulting in the high incidence of cardiovascular diseases, which leads to the most important cause of mortality in the developed world [4–6].

To investigate a possible correlation between this general dysmetabolic condition and specific gene regulation, we previously searched for mRNAs that are differentially expressed from cultured hepatoma cells treated with hypercholesterolaemic sera compared with cells treated with normocholesterolaemic sera [4]. Our data indicated that hypercholesterolaemic sera specifically modulate several mRNAs among them, the most relevant being mitochondrial 3-hydroxy-3-methylglutaryl-coenzyme A synthase (HMGCS2). HMGCS2 is an essential enzyme in the biochemical pathway leading to mitochondrial ketone body production and is already reported in the literature to be involved in the development of several chronic pathologies [5–8].

Extending this study, in the present work, we report an NMR metabolomic analysis of the sera previously used to treat hepatoma cultured cells. Metabolomics is the large-scale study of metabolites within cells, biofluids, tissues, or organisms. As such, metabolomics detects systemic fluctuations of multiple metabolite concentrations in response to drugs, diet, lifestyle, environment, stimuli, and genetic modulations. A variety of compounds (charged, neutral, hydrophobic, hydrophilic) are simultaneously, qualitatively, and quantitatively detected in biologic samples using NMR-based metabolomics analysis [9, 10]. As hypercholesterolaemia reflects metabolic disease that includes but is not limited to lipidic components, the determination of the metabolomics profile is appropriate to produce many points of observation on a complex disease characterized by multifactorial and heterogeneous aspects [6]. Our data show concentration abnormalities of metabolites involved in energy related pathways, indicating that the transcriptional regulation of the gene HMGCS2 affects the synthesis of metabolites involved in mitochondrial energy processes. In particular, samples previously found to be able to increase HMGCS2 mRNA levels in cultured cells also contain higher amounts of metabolites of its encoded enzyme protein product.

## Materials and methods

### Participants

Human serum samples were selected from October 2009 to July 2010 to study serum composition, as previously described [11], from the Clinical Pathology Laboratory, Santa Maria Goretti Hospital, AUSL Latina, Lt, Italy. The study protocol was approved by the institutional ethics committee (Comitato Etico Lazio 2 Azienda Unità Sanitaria Locale Latina), and all subjects gave written informed consent in accordance with the 1964 Helsinki declaration and its later amendments. The normocholesterolaemic group included 16 samples with cholesterolaemia below 170 mg/dl, while the hypercholesterolaemic group included 16 samples with cholesterolaemia above 260 mg/dl. All subjects were healthy males aged between 40 and 50 years. Inclusion criteria included patients presenting abnormal serum cholesterol levels, with no other disease symptoms, and healthy individuals who voluntarily agreed to participate in this study. Exclusion criteria included all subjects presenting any pathological condition or other abnormal serum values, in addition to cholesterol. No access to any identifying participant information was available besides cholesterol concentrations and the absence of other disease symptoms.

### NMR sample preparation

NMR sample preparation and NMR spectra acquisition were performed as previously reported [9, 12]. Blood was collected into standard blood collection tubes and allowed to clot at room temperature for 30 to 120 min before centrifugation.

Serum was aliquoted and stored at -80°C in Greiner cryogenic vials before NMR spectrometry measurements. Before being transferred to a 5-mm heavy-walled NMR tube, samples were thawed at room temperature and then spun at 3000 rpm using a Vivaspin^®^ 6 centrifugal concentrator to remove proteic and particulate matter. Serum supernatant was removed, and to prepare NMR samples, 425 μL of each sample was added to 25 μL of 1 M potassium phosphate buffer (pH 7.4) and 50 μL of D_2_O. Trimethylsilyl propionic-2,2,3,3-d_4_ acid, sodium salt (TSP-d_4_ 0.1% in D_2_O) was used as an internal reference for alignment and quantification of NMR signals; the mixture, homogenized by vortexing for 30 s, was transferred to a 5 mm NMR tube (Bruker NMR tubes) before the analysis started [9].

### NMR spectroscopy and processing

NMR experiments were carried out on a Bruker DRX600 MHz spectrometer equipped with a 5 mm triple-resonance z-gradient CryoProbe. TOPSPIN, version 3.0, was used for spectrometer control and data processing (Bruker Biospin). 1D NOESY experiments [13, 14] were acquired using spectra with 14 ppm, 16 k data points, excitation sculpting for water suppression, 192 transients, 4 s relaxation delay, and 60 ms mixing time. The pulse sequence used included an excitation sculpting routine for the suppression of the water signal [15]. Due to the effect of excitation sculpting on the signal height of resonances in the region close to the water resonance [16, 17], the metabolites that have resonances close to this region (ascorbate, glucose, mannose, and pyroglutamate) were quantified using resonances from those metabolites in other spectral regions. A weighted Fourier transform was applied to the time domain data with a 0.5 Hz line-broadening followed by manual phase and baseline correction in preparation for targeted profiling analysis.

### NMR data analysis

NMR spectra were manually phased and baseline corrected. Quantification of serum metabolites was achieved using Chenomx NMR-Suite v8.0 (Chenomx Inc.). Briefly, the Chenomx profiler is linked to the human metabolome database (HMDB) containing more than 250 metabolite NMR spectral signatures encoded at different spectrometer ^1^H frequencies, including 600 MHz (http://www.hmdb.ca). A comparison of the spectral data obtained for each serum sample with the Chenomx metabolite library results in a list of compounds together with their respective concentrations based on the known concentration of the added internal reference compound, TSP-d_4_.

We submitted our data to the MetaboLights database with ID code MTBLS1416 [18].

### Statistical analysis

Multivariate statistical analysis, principal component analysis (PCA) and partial least-squares discriminant analysis (PLS-DA) were conducted with normalized metabolomics data using MetaboAnalyst 4.0 (http://www.metaboanalyst.ca/) [19]. The performance of the PCA and PLS-DA model was evaluated using the coefficient Q2 (using the 7-fold internal cross-validation method) and the coefficient R2, defining the variance predicted and explained by the model, respectively. The loading plot was used to identify significant metabolites responsible for maximum separation in the PLS-DA score plot, and these metabolites were ranked according to their variable influence on projection (VIP) scores. VIP scores are weighted sums of squares of the PLS-DA weights, which indicate the importance of the variable.

## Results

### Multivariate data analysis

**Fig 1:**
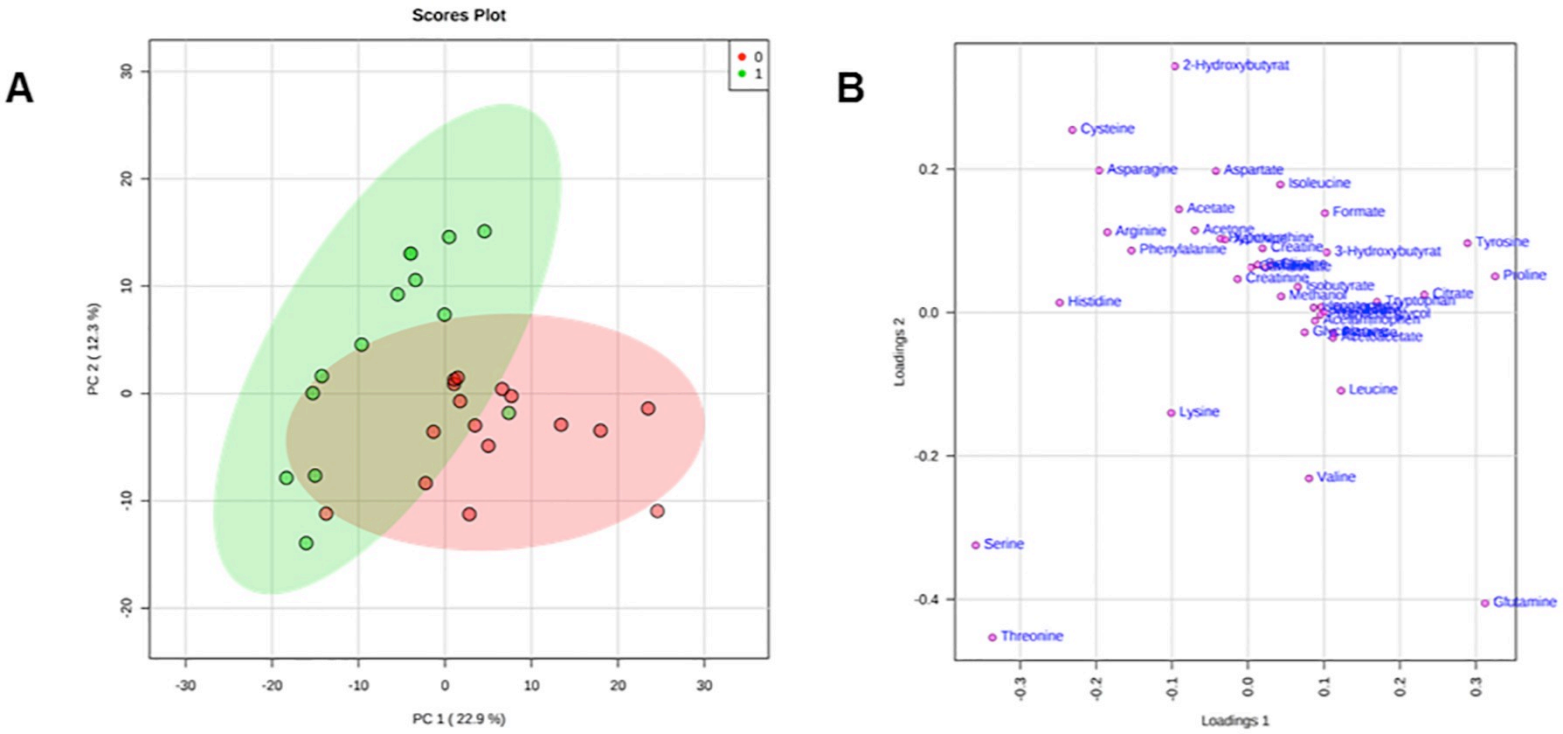
PCA score scatter plot (A) and PCA loading scatter plot (B) for the ^1^H NMR data collected in 1D NOESY spectra acquired at 600 MHz. Data are relative to 14 hypercholesterolaemic (green circles) and 16 normocholesterolaemic (red circles) human sera.

Matrices, including metabolites and their concentrations as derived from ^1^H NMR data collected in 1D NOESY [13, 14], were analysed according to multivariate statistical analysis using MetaboAnalyst 4.0 [19]. Original matrices, including 16 normocholesterolaemic sera and 16 hypercholesterolaemic sera, were normalised according to the concentration ranges of the human metabolome database; as a result, the data of 2 hypercholesterolaemic sera were identified as outliers and excluded [20]. The data matrix, after normalization by sum and *Pareto scaling*, was analysed by PCA (S1 and S2 Figs). To minimize false discoveries and to obtain robust statistical models, *T-test* and *fold change* tests were applied according to good standardized practice (S1 and S2 Tables, S3 and S4 Figs) [19]. For each sample, 41 metabolites were identified and quantified. In Fig 1, PCA shows that the datasets relative to hypercholesterolaemic sera are well separated from those of normocholesterolaemic sera. The first component explains 22.9% of the variance, while the second explains 12.3% (S3 Table).

**Fig 2:**
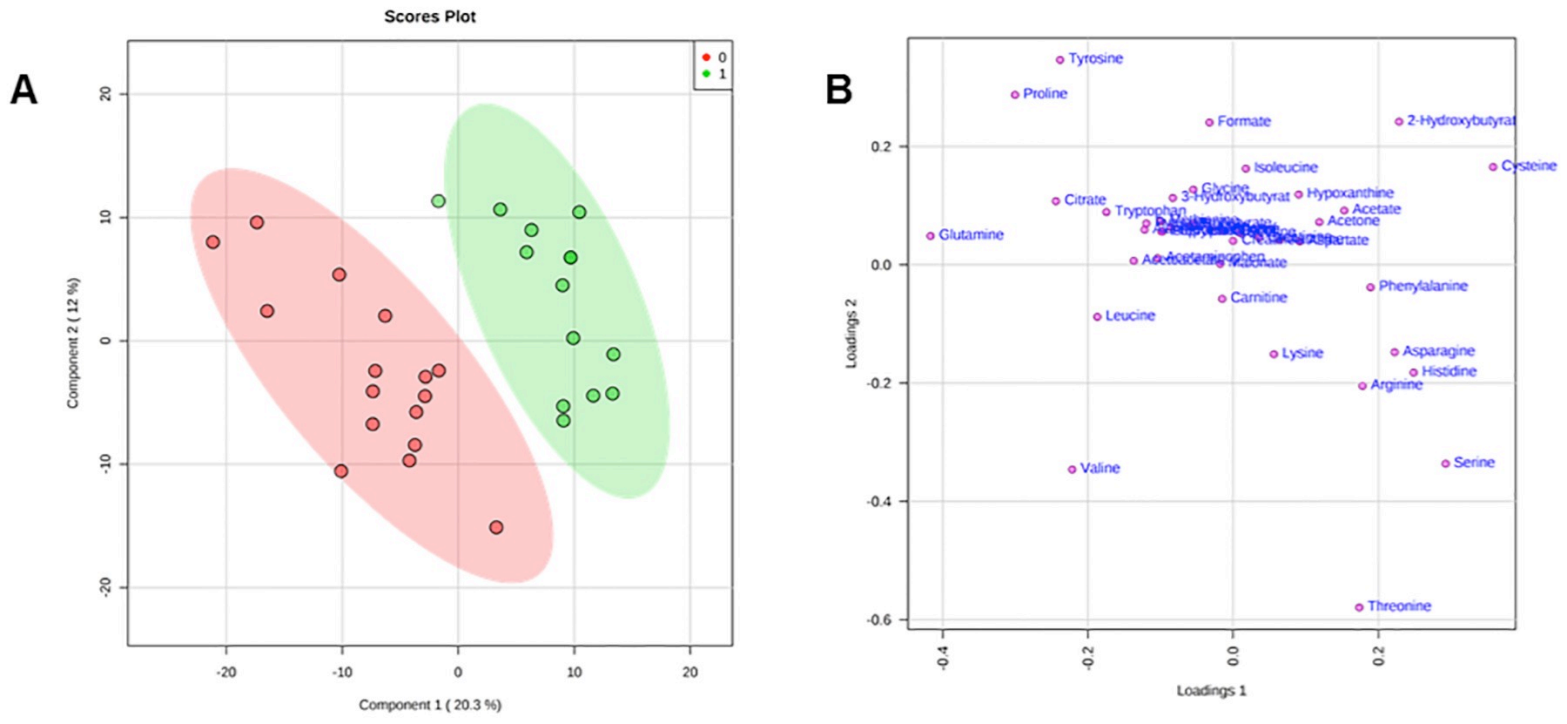
PLS-DA score scatter plot (A) and PLS-DA loading scatter plot (B) for the ^1^H NMR data collected in 1D NOESY spectra acquired at 600 MHz. Data represent 14 hypercholesterolaemic (green circles) and 16 normocholesterolaemic (red circles) human sera.

**Fig 3:**
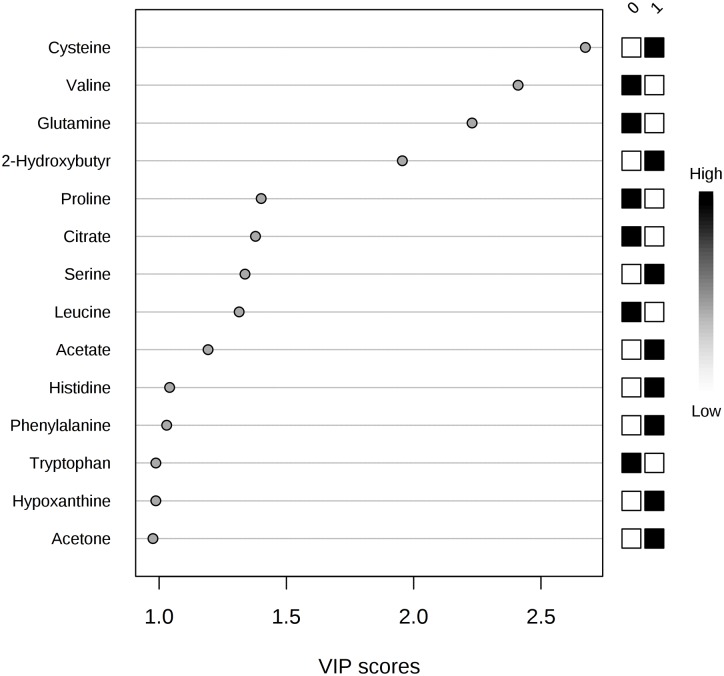
Metabolites discriminating hypercholesterolaemic from normocholesterolaemic samples, according to VIP score values.

To improve the separation observed with the PCA model, supervised PLS-DA was applied: the fitting (R2Y) value was 95.5%, and the predictive (Q2Y) value was 68.7% (S4 Table, S5 Fig). Inspection of the PLS-DA score scatter plot (Fig 2A) and loading scatter plot (Fig 2B) point to 2-hydroxybutyrate, citrate, and acetate as metabolites that significantly discriminate hypercholesterolaemic sera from normocholesterolaemic sera; significant differences in concentration can also be observed for acetone, cysteine, and proline. This evidence is confirmed by applying VIP score analysis (Fig 3, S5 Table). Accordingly, the metabolites characterized by a VIP score higher than 1 are considered good classifiers between the hypercholesterolaemic and normocholesterolaemic groups. In particular, the graph reported in Fig 3 shows that hypercholesterolaemic sera contain higher concentrations of acetate, cysteine, acetone, and 2-hydroxybutyrate and lower concentrations of citrate, glutamine, valine, proline, leucine, and tryptophan than normocholesterolaemic sera. To gain meaningful insight from these data, we applied metabolic pathway analysis using MetaboAnalyst 4.0.

**Fig 4:**
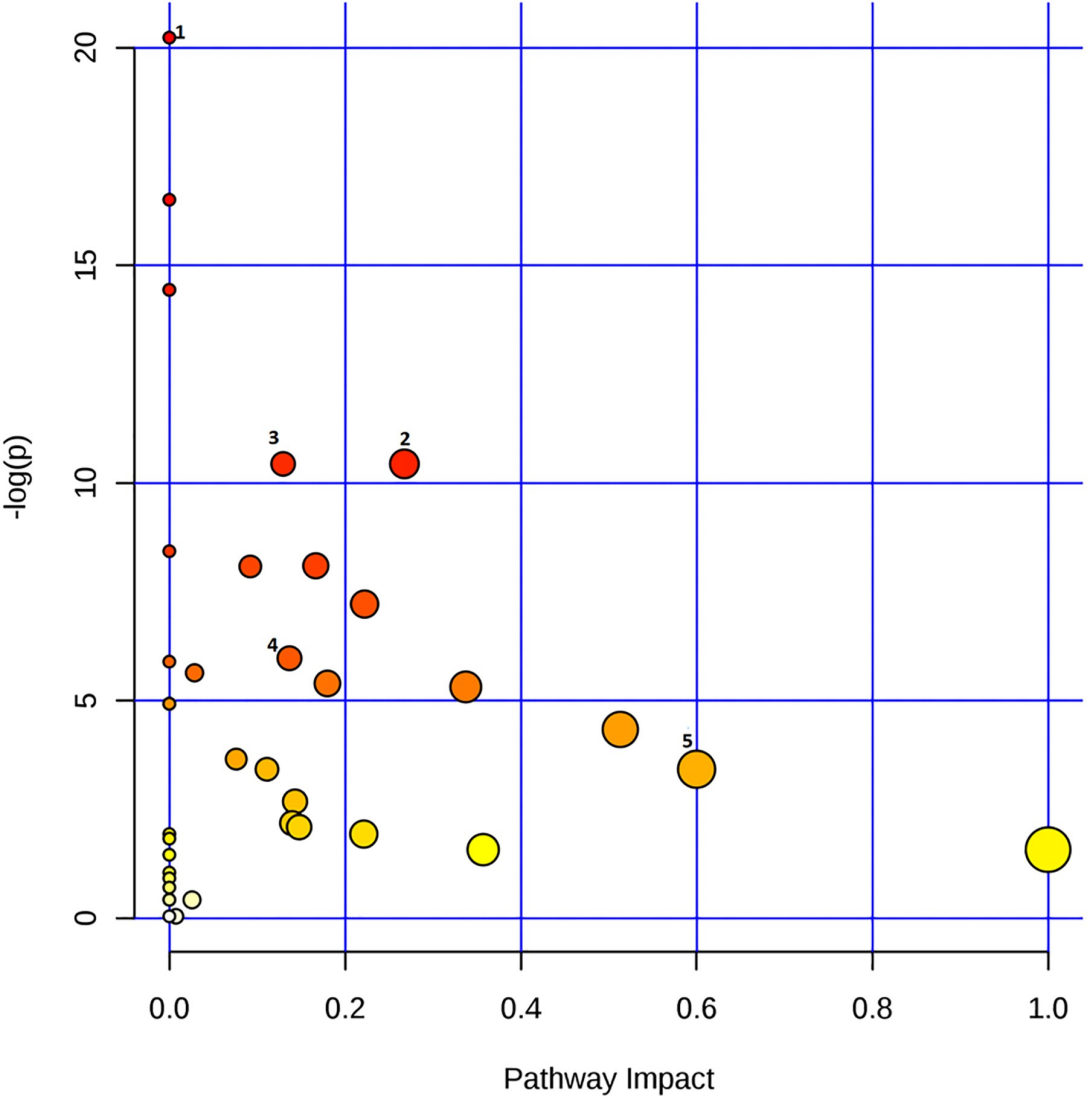
Graph of pathway impact and relative *p*-value. Pathway analysis showing all matched pathways according to *p*-values from pathway enrichment analysis (y-axis) and pathway impact values from pathway topology analysis (x-axis). The colour and size of each circle are based on *p*-values and pathway impact values, respectively. Small *p*-values and large pathway impact circles indicate that the pathway is greatly perturbed. The top 5 pathways in order of *p*-values from the pathway analysis are numbered as follows: 1) pantothenate and CoA biosynthesis, 2) pyruvate metabolism, 3) glycolysis/ gluconeogenesis, 4) citrate cycle (TCA cycle) and 5) synthesis and degradation of ketone bodies.

**Fig 5:**
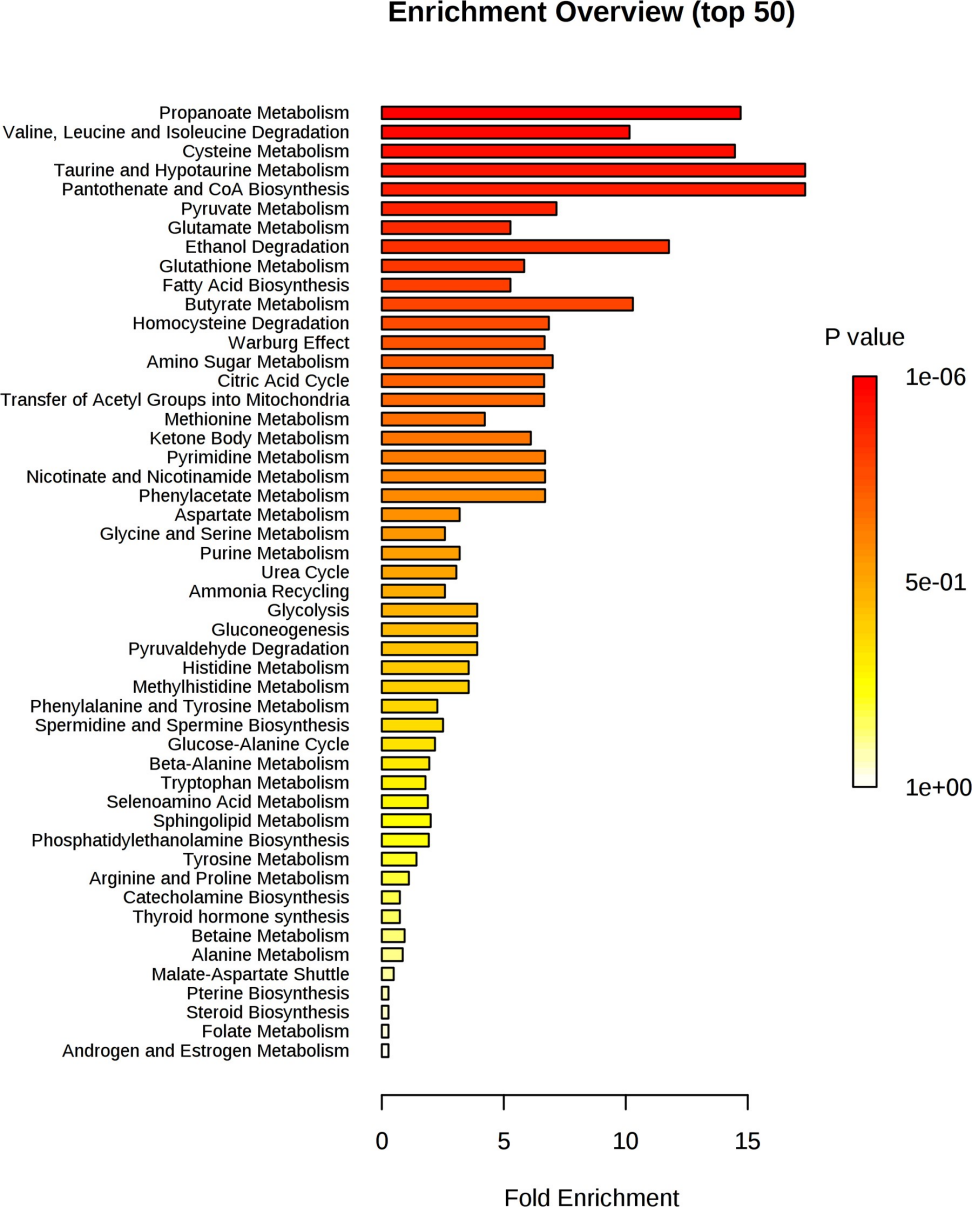
Pathway enrichment analysis: The pathways related to a *p*-value that excludes randomness and correlates with hypercholesterolaemia.

The graph reported in Fig 4 indicates all matched pathways according to *p*-values from pathway enrichment analysis (Fig 5) and according to pathway impact values from pathway topology analysis (S6a–S6e Fig). The pathways were classified according to the total number of compounds found in the KEGG database [21]. Specifically, the metabolic pathways are represented as circles according to their scores from enrichment (*p*-values in the vertical axis) and topology analyses (pathway impact, horizontal axis). Darker circle colours indicate the most significant changes in metabolites in the corresponding pathway. The size of the circle corresponds to the pathway impact score and is correlated with the number of metabolites involved (number of hits). By combining pathways characterized by hits >1 and *p*-values <1e^-6^ (S6 Table), we observed the following top 5 pathways as the most perturbed in our analysis: 1) pantothenate and CoA biosynthesis, 2) pyruvate metabolism, 3) glycolysis/gluconeogenesis, 4) citrate cycle (TCA cycle), and 5) synthesis and degradation of ketone bodies (S6 Fig). The deepening of these results using Reactome analysis [22] indicates that pathways related to mitochondrial dysmetabolism and, in particular, pathways related to the synthesis and metabolism of ketone bodies (*p*-value 0.001) are those most related to the reported metabolic abnormalities. Interestingly, both metabolic pathway analysis and Reactome analysis support the evidence of a relationship between HMGCS2 mRNA overexpression in the hypercholesterolaemic sera and the metabolomic profile characterized by increased levels of acetate and 2-hydroxybutyrate, which are indeed ketone body metabolites.

## Discussion

Analysis of serum components is a traditional source of reference parameters that allows for obtaining relevant diagnostic and prognostic information on numerous diseases. Additionally, the importance of serum composition comes from the fact that it is determined both from endogenous metabolism and from the nutritional intake. Serum components also actively participate in most physiological and pathological mechanisms.

Considering the possibility that serum molecules, including diet-derived nutrients, directly affect pathogenic mechanisms, we previously searched for genes selectively regulated in human hepatoma cells by treatment with dyslipidaemic sera [4]. In these experiments, we found that in response to high cholesterol concentrations, the expression of the HMGCS2 gene was significantly increased [4]. We also observed overexpression of HMGCS2 mRNA [6] in the liver of rats receiving a diet containing only saturated fats [23]. Extending previous works using an NMR-based metabolomics approach, we investigated the hypothesis of a possible correlation between the previously described transcriptomic signature of hepatoma cell lines treated with hypercholesterolaemic sera and the metabolomic signatures of the same sera [4]. In particular, we investigated the hypothesis that the increase in HMGCS2 mRNA levels [6] could affect the activity of its protein product. As a result, our metabolomics data revealed that the hypercholesterolaemic blood sera show a metabolomic profile that is significantly different from that of the normocholesterolaemic sera, where acetate, acetone, 2-hydroxybutyrate, cysteine, valine, and glutamine are the metabolites discriminating the two groups.

The concentrations of 2-hydroxybutyrate are higher in patients with hypercholesterolaemia than in normocholesterolaemic patients.

Dysmetabolism of 2-hydroxybutyrate reflects dysmetabolism of the propanoate pathway. This pathway is one of the most significant pathways identified by the enrichment analysis (Figs 4 and 5), together with the citrate cycle pathway. The propanoate and citrate cycle pathways are both energy-related pathways, and in particular, propanoate is involved in the regulation of the citrate cycle pathway [24].

The concentrations of acetone and acetate are higher in the sera of patients with hypercholesterolaemia. These metabolites are part of the ketone body pathways. Analysis conducted by Reactome confirms the relevance of this pathway in the dysmetabolic framework of subjects with hypercholesterolaemia. Interestingly, this pathway is regulated by HMGCS2, the enzyme whose mRNA we found to be overexpressed in the same hypercholesterolaemia blood sera.

Therefore, our present data support the hypothesis that serum samples previously found to be able to increase HMGCS2 mRNA levels in cultured cells also contain higher amounts of metabolites of its encoded enzyme protein product. The intrinsic enzymatic activity of HMGCS2, which is important within the metabolic pathway to produce ketone bodies, could then be part of the pathogenic mechanisms. These data confirm numerous pieces of evidence from the literature, indicating an important role of HMGCS2 in pathogenic mechanisms. Inhibition of HMGCS2 expression was reported to reduce endothelial damage in a diabetes mellitus model [5]. HMGCS2 deficiencies due to inborn errors of metabolism were associated with dyslipidaemia [7], while HMGCS2 activity was considered to potentially be involved in high cardiovascular risk [8]. Other evidence indicates that the HMGCS2 protein product is a PPARα target [25, 26] since it interacts with PPARα and up-regulates transcription of its gene [27]. HMGCS2 may be a PPARα co-activator [28], while modulating its expression in the cultured HepG2 cell line has complex metabolic effects [29]. Taken together, these data strongly indicate that HMGCS2 expression might have a relevant role within the pathological metabolic condition, which is consequent to inappropriate nutrition and configures an increased cardiovascular risk. The excessive activity of this enzyme could be a consequence of the effect of nutrients and endogenous metabolites acting at the gene regulation level.

Metabolomic analysis indicated increased concentrations of some amino acids, such as cysteine, serine, histidine, and phenylalanine. Previous scientific studies have shown a fundamental role of histidine in the induction of hypercholesterolaemia [24, 30]. Similarly, elevated cysteine levels appear to be related to cardiovascular diseases [31]. Conversely, tryptophan, and leucine have lower concentrations in subjects with hypercholesterolaemia. Tryptophan inhibits gluconeogenesis [32], therefore influencing pyruvate concentrations. The pyruvate pathway is one of the most affected by dyslipidaemic conditions (Fig 4). Many adipose tissues synthesize ketone bodies from leucine, which would explain the reduction in leucine concentration and the increase in fatty acids [33].

According to enrichment pathway analysis, metabolites discriminating the two groups of sera reflect an alteration in the metabolic pathways of pantothenate and CoA biosynthesis, pyruvate, glycolysis/gluconeogenesis, and citrate cycle. While it is not possible to specifically relate these metabolic changes to the pathogenetic mechanisms of clinical conditions associated with hypercholesterolaemia and dyslipidaemia, it is still possible to make some hypotheses. Hypercholesterolaemic and dyslipidaemic conditions leading to cardiovascular conditions are most commonly consequent to a nutritional status with excessive energy and lipid intake. Pantothenate/CoA biosynthesis, pyruvate metabolism, glycolysis/gluconeogenesis, and the citrate cycle are all energy-related pathways. Correspondingly, ketone body pathways are typically increased when excessive energy is derived from lipids. This is consistent with the observed reduction in citrate in hypercholesterolaemic samples, also indicating a decrease in the Krebs cycle, which is the typical metabolic change leading to an increase in ketone body production to process excessive acetyl CoA, which cannot be catabolized [34]. Additionally, observed variations in hypoxanthine and taurine/hypotaurine metabolism are likely part of these disease mechanisms. In particular, hypoxanthine levels are related to the condition of hypercholesterolaemia [35].

## Conclusions

Administration of human hepatoma cells with hypercholestaerolaemic blood sera previously induced an increase in the mRNA expression of HMGCS2, an enzyme involved in the ketone body pathway. In the present work, the sera previously used to treat hepatoma cells have been the object of NMR metabolomics analysis. The data thus obtained show concentration abnormalities of metabolites involved in energy related pathways. Serum samples previously found to be able to increase HMGCS2 mRNA levels in cultured cells also contain higher amounts of acetate and acetoacetate, metabolites of its encoded enzyme protein product.

The data presently reported, integrated by those previously published, define a multi-omics analytical approach to prove that the transcriptional regulation of the gene HMGCS2 has an effect on the synthesis of numerous metabolites related to mitochondrial energy processes.

## Supporting information

S1 Fig Pairwise score plots between the selected PCs. The explained variance of each PC is shown in the corresponding diagonal cell.
(DOC)

S2 Fig Scree plot illustrating the variance explained by PCs. The green line on top shows the accumulated variance explained; the blue line underneath shows the variance explained by individual PC.
(DOC)

S3 Fig Important features selected by fold-change analysis with threshold 2. The red circles represent features above the threshold. The values are on a log scale so that both up-regulated and down- regulated features can be plotted symmetrically.
(DOC)

S4 Fig Important features selected by t-tests with threshold 0.05. The red circles represent features above the threshold. P-values are transformed by -log10 so that the more significant features (with smaller p-values) will be plotted higher on the graph.
(DOC)

S5 Fig PLS-DA classification of the five different components based on the accuracy (blue), R2 (pink), Q2 (light blue). The red star indicates the best classifier.
(DOC)

S6 Fig Pathway viewer: Light blue boxes indicate metabolites (identified by KEGG ID) that are not measured in our data, but are used as background for enrichment analysis; yellow to red colors indicate the metabolites included in our dataset with different levels of significance: **a)** Pantothenate and CoA biosynthesis. C00183 = L-Valine, C0049 = L-Aspartate, C0097 = L-Cysteine. **b)** Pyruvate metabolism. C0022 = Pyruvate, C0033 = Acetate. **c)** Glycolysis / Gluconeogenesis C0033 = Acetate, C00022 = Pyruvate **d)** Citrate cycle (TCA). C00022 = Pyruvate, C00158 = Citrate; Citric acid; 2-Hydroxy-1,2,3-propanetricarboxylic acid; 2-Hydroxytricarballylic acid. **e)** Ketone bodies degradation and synthesis. C01089 = (R)-3-Hydroxybutanoate, C00164 = Acetoacetate.
(DOC)

S1 Table Important features identified by fold change and logarithmic fold change (*log2(FC)*) parameters calculated. (DOC)

S2 Table Important features identified by t-tests values, p-values (threshold <0.05), logarithmic p-values, and False Discovery Rate (FDR) parameters calculated for the most statistically significative compounds. (DOC)

S3 Table Data points related to hypercholesterolemic (P) and normocholesterolemic (D) sample coordinates in 3D space. (DOC)

S4 Table PLS-DA classification of the five different components (*comps*) based on accuracy, R2, Q2. (DOC)

S5 Table Metabolite score values relative to the different components PC1-PC2-PC3-PC4-PC5. (DOC)

S6 Table Detailed pathway analysis results, showing the most influential pathways which discriminate hypercholesterolemic vs. normocholesterolemic sera. The pathway was classified according to: total number of compounds found in KEGG database involved in the pathway (*total Cmpd*), analysed compounds involved in pathway (*Hits*), p-value calculated on the hits (*Raw p*), logarithmic p-value (*-log (p)*), p-value adjusted by Holm-Bonferroni (*Holm adjust*) p-value adjusted using False Discovery Rate (*FDR*), the pathway impact value calculated from pathway topology analysis (*Impact*).
(DOC)
