# Supplementary material for: NMR-based metabolomic profile of hypercholesterolemic human sera: Relationship with in vitro gene expression?
Source: PLoS One. 2020 Apr 16;15(4):e0231506. doi: 10.1371/journal.pone.0231506 (PMC7162471; doi:10.1371/journal.pone.0231506)
Supplement: S1 Fig — The explained variance of each PC is shown in the corresponding diagonal cell. (DOC) [file pone.0231506.s001.doc]

**Figure S1:** Pairwise score plots between the selected PCs. The explained variance of each PC is shown in the corresponding diagonal cell.

**
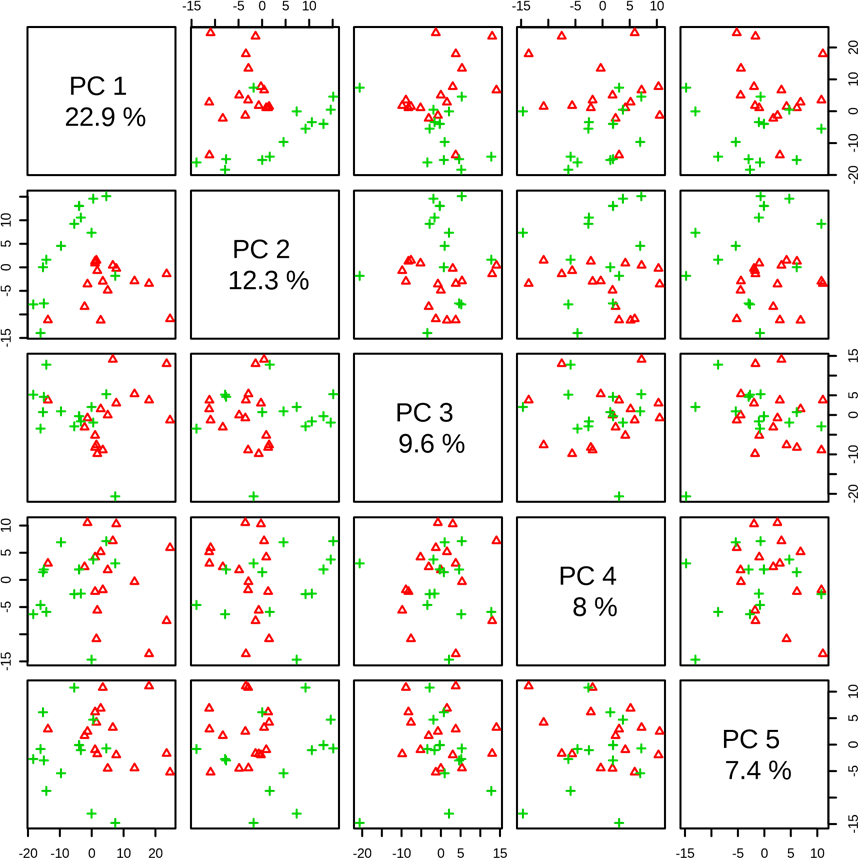
**
