# Supplementary material for: NMR-based metabolomic profile of hypercholesterolemic human sera: Relationship with in vitro gene expression?
Source: PLoS One. 2020 Apr 16;15(4):e0231506. doi: 10.1371/journal.pone.0231506 (PMC7162471; doi:10.1371/journal.pone.0231506)
Supplement: S2 Fig — The green line on top shows the accumulated variance explained; the blue line underneath shows the variance explained by individual PC. (DOC) [file pone.0231506.s002.doc]

**Figure S2:** Scree plot illustrating the variance explained by PCs. The green line on top shows the accumulated variance explained; the blue line underneath shows the variance explained by individual PC.
