# Supplementary material for: NMR-based metabolomic profile of hypercholesterolemic human sera: Relationship with in vitro gene expression?
Source: PLoS One. 2020 Apr 16;15(4):e0231506. doi: 10.1371/journal.pone.0231506 (PMC7162471; doi:10.1371/journal.pone.0231506)

**Figure S3:** Important features selected by fold-change analysis with threshold 2. The red circles represent features above the threshold. The values are on a log scale so that both up-regulated and down- regulated features can be plotted symmetrically.


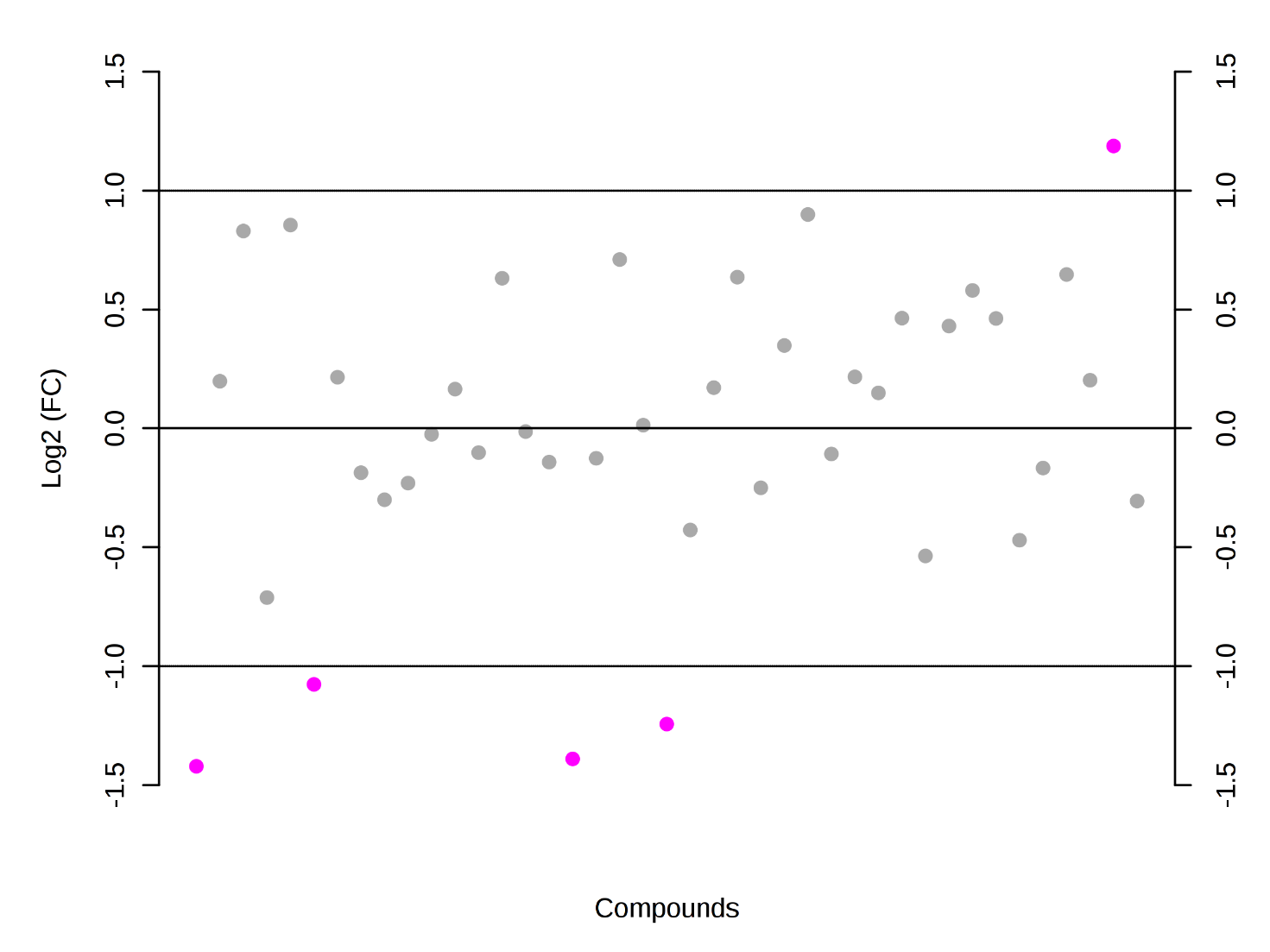

Supplement: S3 Fig — The red circles represent features above the threshold. The values are on a log scale so that both up-regulated and down- regulated features can be plotted symmetrically. (DOC) [file pone.0231506.s003.doc]
