# Supplementary material for: NMR-based metabolomic profile of hypercholesterolemic human sera: Relationship with in vitro gene expression?
Source: PLoS One. 2020 Apr 16;15(4):e0231506. doi: 10.1371/journal.pone.0231506 (PMC7162471; doi:10.1371/journal.pone.0231506)

**Figure S4:** Important features selected by t-tests with threshold 0.05. The red circles represent features above the threshold. P-values are transformed by -log10 so that the more significant features (with smaller p-values) will be plotted higher on the graph.


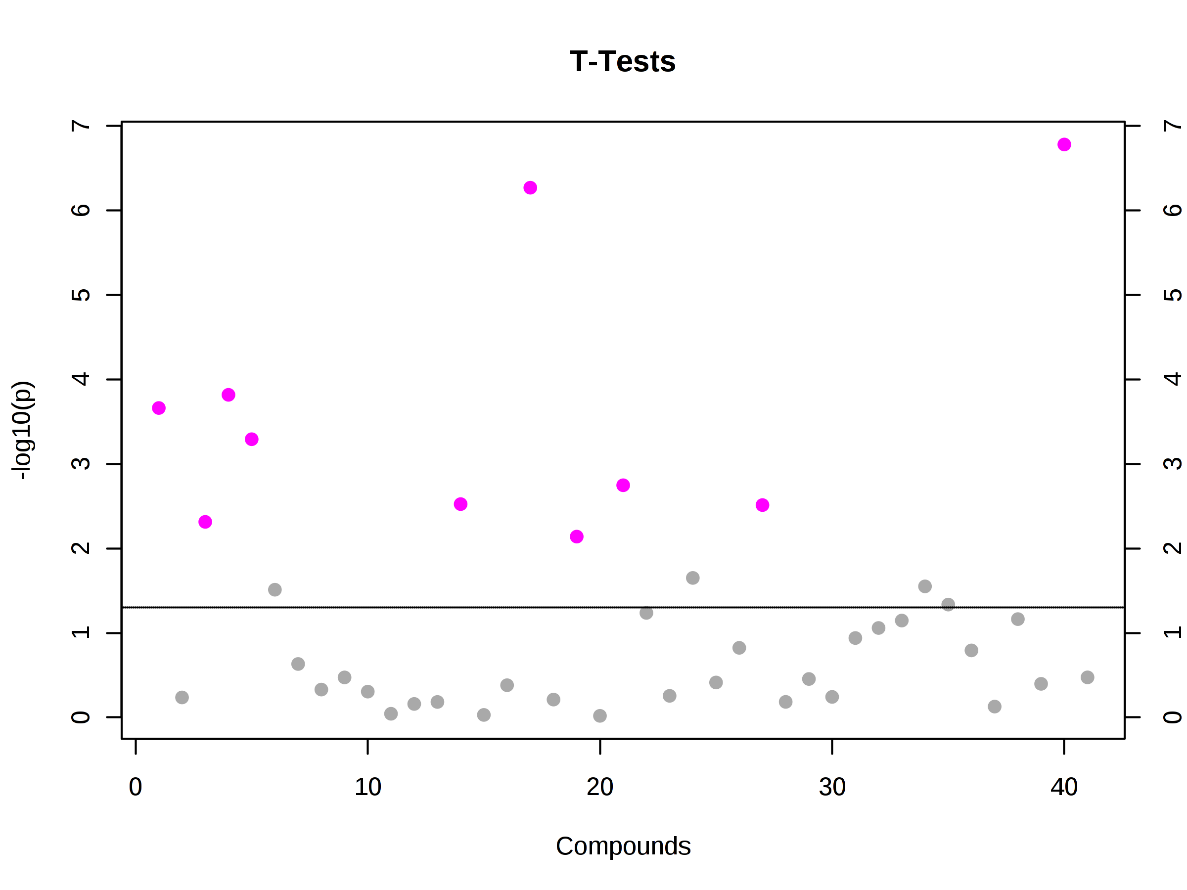

Supplement: S4 Fig — The red circles represent features above the threshold. P-values are transformed by -log10 so that the more significant features (with smaller p-values) will be plotted higher on the graph. (DOC) [file pone.0231506.s004.doc]
