# Supplementary material for: NMR-based metabolomic profile of hypercholesterolemic human sera: Relationship with in vitro gene expression?
Source: PLoS One. 2020 Apr 16;15(4):e0231506. doi: 10.1371/journal.pone.0231506 (PMC7162471; doi:10.1371/journal.pone.0231506)
Supplement: S5 Fig — The red star indicates the best classifier. (DOC) [file pone.0231506.s005.doc]

**Figure S5:** PLS-DA classification of the five different components based on the accuracy (blue), R2 (pink), Q2 (light blue).The red star indicates the best classifier**.**

Accuracy

R2

Q2
