# Supplementary material for: NMR-based metabolomic profile of hypercholesterolemic human sera: Relationship with in vitro gene expression?
Source: PLoS One. 2020 Apr 16;15(4):e0231506. doi: 10.1371/journal.pone.0231506 (PMC7162471; doi:10.1371/journal.pone.0231506)
Supplement: S6 Fig — Pathway viewer: Light blue boxes indicate metabolites (identified by KEGG ID) that are not measured in our data, but are used as background for enrichment analysis; yellow to red colors indicate the metabolites included in our dataset with different levels of significance: a) Pantothenate and CoA biosynthesis. C00183 = L-Valine, C0049 = L-Aspartate, C0097 = L-Cysteine. b) Pyruvate metabolism. C0022 = Pyruvate, C0033 = Acetate. c) Glycolysis / Gluconeogenesis C0033 = Acetate, C00022 = Pyruvate d) Citrate cycle (TCA). C00022 = Pyruvate, C00158 = Citrate; Citric acid; 2-Hydroxy-1,2,3-propanetricarboxylic acid; 2-Hydroxytricarballylic acid. e) Ketone bodies degradation and synthesis. C01089 = (R)-3-Hydroxybutanoate, C00164 = Acetoacetate. (DOC) [file pone.0231506.s006.doc]

**Figure S6:** Pathway viewer: light blue boxes indicate metabolites (identified by KEGG ID) that are not measured in our data, but are used as background for enrichment analysis; yellow to red colors indicate the metabolites included in our dataset with different levels of significance: **a)**  **Pantothenate and CoA biosynthesis**. C00183=L-Valine, C0049=L-Aspartate, C0097=L-Cysteine. **b) Pyruvate metabolism.** C0022=Pyruvate, C0033=Acetate. **c)** **Glycolysis / Gluconeogenesis** C0033=Acetate, C00022=Pyruvate **d) Citrate cycle (TCA).** C00022=Pyruvate, C00158=Citrate; Citric acid; 2-Hydroxy-1,2,3-propanetricarboxylic acid; 2-Hydroxytricarballylic acid. **e) Ketone bodies degradation and synthesis.** C01089=(R)-3-Hydroxybutanoate, C00164=Acetoacetate.

**
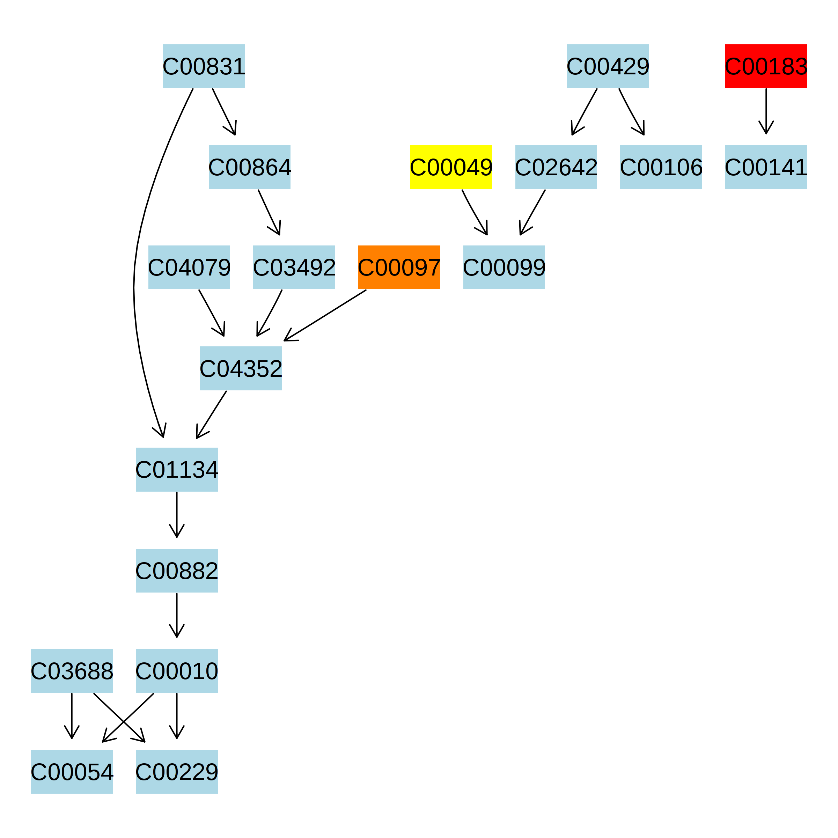

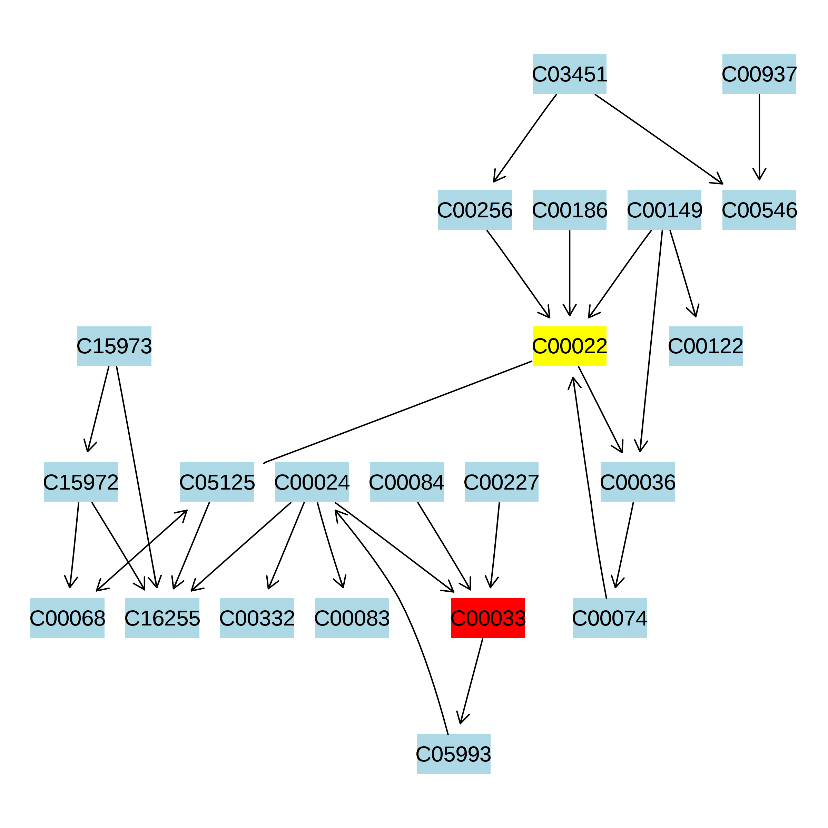
**

**a)**

**b)**


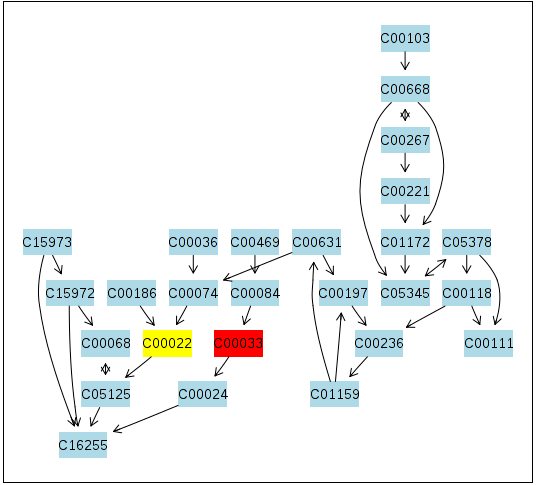

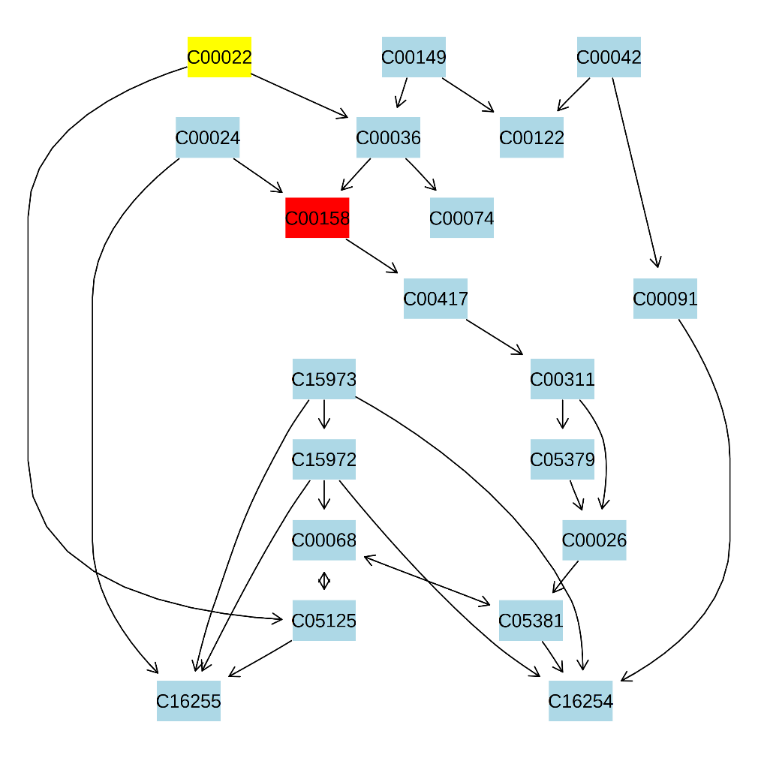


**c)**

**d)**


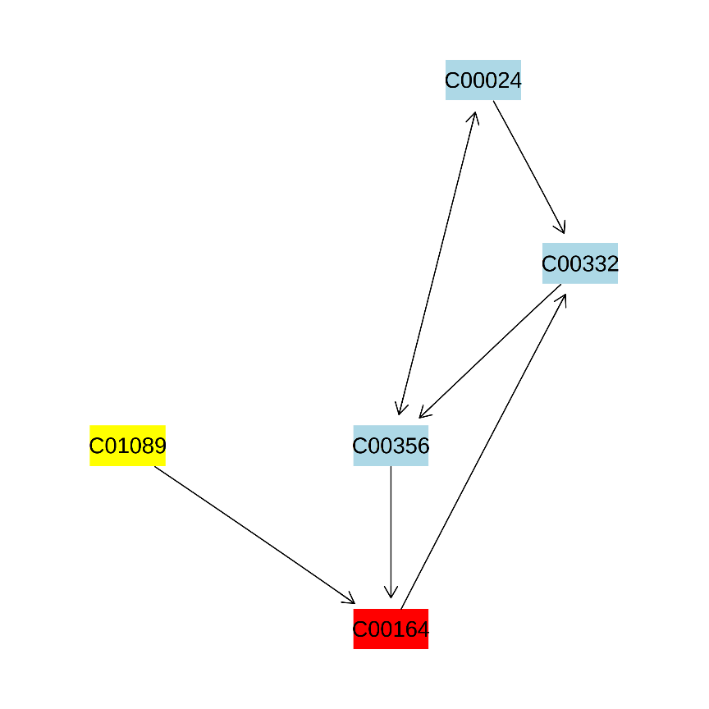


**e)**
