# Supplementary material for: NMR-based metabolomic profile of hypercholesterolemic human sera: Relationship with in vitro gene expression?
Source: PLoS One. 2020 Apr 16;15(4):e0231506. doi: 10.1371/journal.pone.0231506 (PMC7162471; doi:10.1371/journal.pone.0231506)
Supplement: S1 Table — (DOC) [file pone.0231506.s007.doc]

**Table S1:** Important features identiﬁed by fold change and logarithmic Fold Change (*log2(FC)*) parameters calculated.

| **Compounds** | **Fold Change** | **log2(FC)** |
| --- | --- | --- |
| 2-Hydroxybutyrate | 0.37365 | -1.4203 |
| Cysteine | 0.38176 | -1.3893 |
| Hypoxanthine | 0.42253 | -1.2429 |
| Valine | 2.2768 | 1.187 |
| Acetone | 0.47434 | -1.076 |
