# Supplementary material for: NMR-based metabolomic profile of hypercholesterolemic human sera: Relationship with in vitro gene expression?
Source: PLoS One. 2020 Apr 16;15(4):e0231506. doi: 10.1371/journal.pone.0231506 (PMC7162471; doi:10.1371/journal.pone.0231506)
Supplement: S2 Table — (DOC) [file pone.0231506.s008.doc]

**Table S2:** Important features identiﬁed by t-tests values, p-values (Threshold <0.05), logarithmic p-values, and false discovery rate (FDR) parameters calculated for the most statistically significative compounds.

| **Compounds** | **t.stat** | **p.value** | **-log10(p)** | **FDR** |
| --- | --- | --- | --- | --- |
| Valine | 6.9069 | 1.656e-07 | 6.7809 | 6.7895e-06 |
| Cysteine | -6.4586 | 5.3765e-07 | 6.2695 | 1.1022e-05 |
| Acetate | -4.3765 | 0.00015201 | 3.8181 | 0.0020775 |
| 2-Hydroxybutyrate | -4.2441 | 0.00021771 | 3.6621 | 0.0022316 |
| Acetoacetate | 3.9278 | 0.00050979 | 3.2926 | 0.0041803 |
| Hypoxanthine | -3.4516 | 0.0017873 | 2.7478 | 0.012213 |
| Citrate | 3.2516 | 0.0029875 | 2.5247 | 0.01572 |
| Leucine | 3.2412 | 0.0030673 | 2.5133 | 0.01572 |
| Acetaminophen | 3.0592 | 0.004849 | 2.3143 | 0.02209 |
| Glutamine | 2.8966 | 0.0072435 | 2.1401 | 0.029698 |
