# Supplementary material for: NMR-based metabolomic profile of hypercholesterolemic human sera: Relationship with in vitro gene expression?
Source: PLoS One. 2020 Apr 16;15(4):e0231506. doi: 10.1371/journal.pone.0231506 (PMC7162471; doi:10.1371/journal.pone.0231506)
Supplement: S3 Table — (DOC) [file pone.0231506.s009.doc]

**Table S3:** Data points related to hypercholesterolemic (P) and normocholesterolemic (D) sample coordinates in 3D space.

| **Samples** | **PC1** | **PC2** | **PC3** |
| --- | --- | --- | --- |
| P1 | -14.247 | 1.6244 | 12.782 |
| P2 | -15.296 | 0.027499 | 0.7398 |
| P3 | -5.4817 | 9.2336 | -2.8755 |
| P4 | -18.347 | -7.8686 | 5.1617 |
| P5 | -0.055791 | 7.3503 | 2.042 |
| P6 | 0.47468 | 14.586 | -1.9081 |
| P7 | 4.5715 | 15.117 | 5.2911 |
| P8 | -9.6404 | 4.5531 | 0.94662 |
| P9 | 7.3814 | -1.8108 | -20.665 |
| P10 | -16.059 | -13.95 | -3.4478 |
| P11 | -15.031 | -7.658 | 4.6126 |
| P12 | -3.4157 | 10.576 | -1.6197 |
| P14 | -3.9663 | 13.043 | -0.8933 |
| D1 | 17.988 | -3.4538 | 3.7713 |
| D2 | 13.426 | -2.906 | 5.3411 |
| D3 | 24.572 | -10.956 | -1.3015 |
| D4 | 23.499 | -1.3955 | 13.008 |
| D5 | 2.8165 | -11.254 | 1.5267 |
| D6 | 6.6173 | 0.41353 | 14.094 |
| D7 | 7.6895 | -0.24584 | 2.9956 |
| D8 | 1.0466 | 0.87775 | -5.1929 |
| D9 | -2.2467 | -8.3549 | -3.1028 |
| D10 | -13.752 | -11.202 | 3.7476 |
| D11 | -1.337 | -3.5677 | -0.77357 |
| D12 | 5.0138 | -4.8835 | -0.024943 |
| D13 | 1.0599 | 1.2795 | -8.2541 |
| D14 | 1.4672 | 1.4823 | -76101 |
| D15 | 1.7462 | -0.71756 | -9.8381 |
| D16 | 3.4715 | -2.9796 | -8.8969 |
