# Supplementary material for: NMR-based metabolomic profile of hypercholesterolemic human sera: Relationship with in vitro gene expression?
Source: PLoS One. 2020 Apr 16;15(4):e0231506. doi: 10.1371/journal.pone.0231506 (PMC7162471; doi:10.1371/journal.pone.0231506)
Supplement: S4 Table — (DOC) [file pone.0231506.s010.doc]

**Table S4****:** PLS-DA classification of the five different components (*comps*) based on accuracy, R2, Q2.

| **Measure** | **1 comps** | **2 comps** | **3 comps** | **4 comps** | **5 comps** |
| --- | --- | --- | --- | --- | --- |
| **Accuracy** | 0.88636 | 0.97277 | 0.93182 | 0.97277 | 0.97277 |
| **R2** | 0.69915 | 0.8722 | 0.93255 | 0.95638 | 0.96857 |
| **Q2** | 0.51007 | 0.60993 | 0.66699 | 0.68767 | 0.57122 |
