# Supplementary material for: NMR-based metabolomic profile of hypercholesterolemic human sera: Relationship with in vitro gene expression?
Source: PLoS One. 2020 Apr 16;15(4):e0231506. doi: 10.1371/journal.pone.0231506 (PMC7162471; doi:10.1371/journal.pone.0231506)
Supplement: S5 Table — (DOC) [file pone.0231506.s011.doc]

**Table S5:** Metabolite score values relative to the different components PC1-PC2-PC3-PC4-PC5.

| **Metabolite** | **Comp. 1** | **Comp. 2** | **Comp. 3** | **Comp. 4** | **Comp. 5** |
| --- | --- | --- | --- | --- | --- |
| 2-Hydroxybutyrate | 19.555 | 18.409 | 17.911 | 17.737 | 17.635 |
| 3-Hydroxybutyrate | 0.22706 | 0.41462 | 0.40442 | 0.44077 | 0.44954 |
| Acetaminophen | 0.74936 | 0.67603 | 0.67453 | 0.67328 | 0.68825 |
| Acetate | 11.928 | 10.943 | 10.617 | 10.482 | 10.426 |
| Acetoacetate | 0.90625 | 0.80988 | 0.78851 | 0.77876 | 0.77388 |
| Acetone | 0.97646 | 0.90886 | 0.88357 | 0.87895 | 0.87369 |
| Alanine | 0.62995 | 0.59033 | 0.57272 | 0.56545 | 0.56323 |
| Arginine | 0.5714 | 0.84705 | 0.82817 | 0.87651 | 0.87753 |
| Asparagine | 0.63389 | 10.953 | 12.298 | 1.217 | 12.189 |
| Aspartate | 0.40806 | 0.42153 | 0.56812 | 0.56956 | 0.57066 |
| Betaine | 0.043872 | 0.042606 | 0.2121 | 0.25062 | 0.26756 |
| Carnitine | 0.20111 | 0.21847 | 0.21783 | 0.39242 | 0.4097 |
| Choline | 0.087422 | 0.21765 | 0.21559 | 0.27168 | 0.27202 |
| Citrate | 1.379 | 12.505 | 1.218 | 12.144 | 12.068 |
| Creatine | 0.025123 | 0.037625 | 0.11108 | 0.13459 | 0.16077 |
| Creatinine | 0.26709 | 0.24234 | 0.25814 | 0.27264 | 0.27251 |
| Cysteine | 26.741 | 2.43 | 23.574 | 23.287 | 2.314 |
| Formate | 0.25251 | 0.59174 | 0.60111 | 0.596 | 0.61282 |
| Glutamine | 22.293 | 20.575 | 20.484 | 20.479 | 20.351 |
| Glycine | 0.042252 | 0.36871 | 0.35766 | 0.38268 | 0.44926 |
| Hypoxanthine | 0.98772 | 10.068 | 0.99181 | 0.99791 | 10.028 |
| Histidine | 10.419 | 11.365 | 11.132 | 1.115 | 11.102 |
| Isobutyrate | 0.15613 | 0.26735 | 0.26165 | 0.28964 | 0.29553 |
| Isopropanol | 0.51181 | 0.47863 | 0.46491 | 0.46257 | 0.45967 |
| Isoleucine | 0.41873 | 0.52178 | 0.52195 | 0.51908 | 0.54024 |
| Isovalerate | 0.35603 | 0.39117 | 0.38335 | 0.38862 | 0.38617 |
| Leucine | 13.148 | 11.819 | 1.155 | 11.692 | 1.166 |
| Lysine | 0.27101 | 0.26388 | 0.47487 | 0.47384 | 0.48409 |
| Malonate | 0.2838 | 0.32352 | 0.40084 | 0.39592 | 0.3944 |
| Methanol | 0.19956 | 0.17818 | 0.26919 | 0.34804 | 0.36046 |
| Methionine | 0.477 | 0.46639 | 0.45323 | 0.4476 | 0.4448 |
| Phenylalanine | 10.303 | 0.94486 | 0.92549 | 0.91707 | 0.9688 |
| Proline | 14.013 | 13.962 | 13.779 | 13.664 | 13.579 |
| Propyleneglycol | 0.60178 | 0.53789 | 0.54698 | 0.5515 | 0.54825 |
| Pyruvate | 0.66106 | 0.60295 | 0.59256 | 0.60894 | 0.61877 |
| Serine | 13.378 | 13.535 | 13.735 | 14.237 | 14.207 |
| Threonine | 0.30864 | 0.99381 | 12.965 | 12.844 | 12.819 |
| Tryptophan | 0.98784 | 0.89526 | 0.87739 | 0.87063 | 0.86839 |
| Tyrosine | 0.65106 | 11.905 | 11.558 | 11.413 | 11.432 |
| Valine | 24.099 | 24.517 | 23.879 | 23.597 | 23.508 |
| Xanthine | 0.38401 | 0.34435 | 0.38154 | 0.37856 | 0.3914 |
