# Supplementary material for: NMR-based metabolomic profile of hypercholesterolemic human sera: Relationship with in vitro gene expression?
Source: PLoS One. 2020 Apr 16;15(4):e0231506. doi: 10.1371/journal.pone.0231506 (PMC7162471; doi:10.1371/journal.pone.0231506)
Supplement: S6 Table — The pathway was classified according to: total number of compounds found in KEGG database involved in the pathway (total Cmpd), analysed compounds involved in pathway (Hits), p-value calculated on the hits (Raw p), logarithmic p-value (-log (p)), p-value adjusted by Holm-Bonferroni (Holm adjust) p-value adjusted using False Discovery Rate (FDR), the pathway impact value calculated from pathway topology analysis (Impact). (DOC) [file pone.0231506.s012.doc]

**Table S6:** Detailed Pathway Analysis results, showing the most influential pathways which discriminate hypercholesterolemic vs. normocholesterolemic sera. The pathway was classified according to: total number of compounds found in KEGG database involved in the pathway (*total Cmpd*), analysed compounds involved in pathway (*Hits*), p-value calculated on the hits (*Raw p*), logarithmic p-value (*-log (p)*), p-value adjusted by Holm-Bonferroni (*Holm adjust*) p-value adjusted using False Discovery Rate (*FDR*), the pathway impact value calculated from pathway topology analysis (*Impact*).

| **Pathway** | **Total Cmpd** | **Hits** | **Raw p** | **-log(p)** | **Holm adjust** | **FDR** | **Impact** |
| --- | --- | --- | --- | --- | --- | --- | --- |
| Pantothenate and CoA biosynthesis | 19 | 3 | 1.64E-09 | 2.02E+01 | 6.39E-08 | 6.39E-08 | 0.00 |
| Valine, leucine and isoleucine degradation | 40 | 4 | 6.79E-08 | 1.65E+01 | 2.58E-06 | 1.32E-06 | 0.00 |
| Taurine and hypotaurine metabolism | 8 | 1 | 5.38E-07 | 1.44E+01 | 1.99E-05 | 5.24E-06 | 0.00 |
| Thiamine metabolism | 7 | 1 | 5.38E-07 | 1.44E+01 | 1.99E-05 | 5.24E-06 | 0.00 |
| Pyruvate metabolism | 22 | 2 | 2.93E-05 | 1.04E+01 | 1.03E-03 | 1.90E-04 | 0.27 |
| Glycolysis / Gluconeogenesis | 26 | 2 | 2.93E-05 | 1.04E+01 | 1.03E-03 | 1.90E-04 | 0.13 |
| Propanoate metabolism | 23 | 1 | 2.18E-04 | 8.43E+00 | 7.18E-03 | 1.21E-03 | 0.00 |
| Aminoacyl-tRNA biosynthesis | 48 | 19 | 3.05E-04 | 8.10E+00 | 9.75E-03 | 1.34E-03 | 0.17 |
| Glutathione metabolism | 28 | 2 | 3.09E-04 | 8.08E+00 | 9.75E-03 | 1.34E-03 | 0.09 |
| Cysteine and methionine metabolism | 33 | 4 | 7.34E-04 | 7.22E+00 | 2.20E-02 | 2.86E-03 | 0.22 |
| Citrate cycle (TCA cycle) | 20 | 2 | 2.55E-03 | 5.97E+00 | 7.39E-02 | 8.95E-03 | 0.14 |
| Valine, leucine and isoleucine biosynthesis | 8 | 4 | 2.75E-03 | 5.89E+00 | 7.71E-02 | 8.95E-03 | 0.00 |
| Purine metabolism | 65 | 3 | 3.56E-03 | 5.64E+00 | 9.61E-02 | 1.07E-02 | 0.03 |
| Glyoxylate and dicarboxylate Metabolism | 32 | 7 | 4.55E-03 | 5.39E+00 | 1.18E-01 | 1.27E-02 | 0.18 |
| Alanine, aspartate and glutamate metabolism | 28 | 6 | 4.92E-03 | 5.31E+00 | 1.23E-01 | 1.28E-02 | 0.34 |
| Pyrimidine metabolism | 39 | 1 | 7.24E-03 | 4.93E+00 | 1.74E-01 | 1.57E-02 | 0.00 |
| D-Glutamine and D-glutamate metabolism | 6 | 1 | 7.24E-03 | 4.93E+00 | 1.74E-01 | 1.57E-02 | 0.00 |
| Nitrogen metabolism | 6 | 1 | 7.24E-03 | 4.93E+00 | 1.74E-01 | 1.57E-02 | 0.00 |
| Glycine, serine and threonine metabolism | 33 | 8 | 1.31E-02 | 4.34E+00 | 2.75E-01 | 2.68E-02 | 0.51 |
| Arginine biosynthesis | 14 | 3 | 2.59E-02 | 3.65E+00 | 5.17E-01 | 5.05E-02 | 0.08 |
| Synthesis and degradation of ketone bodies | 5 | 2 | 3.27E-02 | 3.42E+00 | 6.21E-01 | 5.79E-02 | 0.60 |
| Butanoate metabolism | 15 | 2 | 3.27E-02 | 3.42E+00 | 6.21E-01 | 5.79E-02 | 0.11 |
| Tryptophan metabolism | 41 | 1 | 6.86E-02 | 2.68E+00 | 1.00E+00 | 1.16E-01 | 0.14 |
| Tyrosine metabolism | 42 | 3 | 1.13E-01 | 2.18E+00 | 1.00E+00 | 1.83E-01 | 0.14 |
| Arginine and proline metabolism | 38 | 4 | 1.23E-01 | 2.09E+00 | 1.00E+00 | 1.93E-01 | 0.15 |
| Histidine metabolism | 16 | 2 | 1.45E-01 | 1.93E+00 | 1.00E+00 | 2.09E-01 | 0.22 |
| beta-Alanine metabolism | 21 | 2 | 1.45E-01 | 1.93E+00 | 1.00E+00 | 2.09E-01 | 0.00 |
| Sphingolipid metabolism | 21 | 1 | 1.61E-01 | 1.83E+00 | 1.00E+00 | 2.24E-01 | 0.00 |
| Phenylalanine, tyrosine and tryptophan biosynthesis | 4 | 2 | 2.07E-01 | 1.57E+00 | 1.00E+00 | 2.69E-01 | 1.00 |
| Phenylalanine metabolism | 10 | 2 | 2.07E-01 | 1.57E+00 | 1.00E+00 | 2.69E-01 | 0.36 |
| Selenocompound metabolism | 20 | 1 | 2.33E-01 | 1.46E+00 | 1.00E+00 | 2.93E-01 | 0.00 |
| Fatty acid biosynthesis | 47 | 1 | 3.51E-01 | 1.05E+00 | 1.00E+00 | 4.28E-01 | 0.00 |
| Ubiquinone and other terpenoid-quinone biosynthesis | 9 | 1 | 4.01E-01 | 9.15E-01 | 1.00E+00 | 4.73E-01 | 0.00 |
| Nicotinate and nicotinamide metabolism | 15 | 1 | 4.95E-01 | 7.03E-01 | 1.00E+00 | 5.68E-01 | 0.00 |
| Lysine degradation | 25 | 1 | 6.55E-01 | 4.23E-01 | 1.00E+00 | 6.92E-01 | 0.00 |
| Biotin metabolism | 10 | 1 | 6.55E-01 | 4.23E-01 | 1.00E+00 | 6.92E-01 | 0.00 |
| Glycerophospholipid metabolism | 36 | 1 | 6.56E-01 | 4.21E-01 | 1.00E+00 | 6.92E-01 | 0.03 |
| Primary bile acid biosynthesis | 46 | 1 | 9.58E-01 | 4.29E-02 | 1.00E+00 | 9.58E-01 | 0.01 |
| Porphyrin and chlorophyll metabolism | 30 | 1 | 9.58E-01 | 4.29E-02 | 1.00E+00 | 9.58E-01 | 0.00 |
